# A Multiport Network-Based Integrated Sensing System Using Rectangular Cavity Resonators for Volatile Organic Compounds

**DOI:** 10.3390/s26010189

**Published:** 2025-12-27

**Authors:** Haoxiang Wang, Jie Huang

**Affiliations:** College of Engineering and Technology, Southwest University, Chongqing 400715, China; a1393731014@email.swu.edu.cn

**Keywords:** rectangular cavity resonators, multiport network, volatile gas, six-port reflectometer, temperature and humidity effect

## Abstract

This work presents a novel microwave sensor system for volatile gas detection, integrating sensing elements based on rectangular cavity resonators (RCR) and multiport demodulation circuitry. Initially, a pump-through gas sensing element utilizing an RCR was developed, and its core sensing functionality was experimentally validated. Subsequently, a rat-race coupler was employed to seamlessly integrate two such rectangular cavity resonator elements—serving as reference and sensing branches—within the multiport demodulation network. This configuration enabled an in-depth investigation of the network’s operating principle, elucidating the critical relationship between the reference and sensing arms. The demodulation network translates the critical output phase shift into corresponding power readings. The quantitative relationship linking phase shift to power output was rigorously characterized and utilized as the basis for estimating volatile gas concentration. Finally, a dedicated LabVIEW-based platform was developed for real-time, quantitative volatile gas monitoring. This integrated measurement system demonstrates excellent detection limits (300 ppm for acetone, 200 ppm for ethanol) and exhibits robust mitigation of measurement artifacts caused by ambient temperature and humidity fluctuations. Comprehensive theoretical analysis and experimental results jointly validate the efficacy of the proposed multiport network and RCR volatile gas sensing architecture.

## 1. Introduction

Volatile organic compounds (VOCs) refer to chemical compounds that readily undergo the volatilization process at room temperature. These substances originate from a wide array of sources and represent a highly prevalent category of hazardous chemicals [1,2]. The volatilization process of VOCs leads to a modification of the surrounding gas environment. This environmental modification, in turn, poses potential impacts to various sectors, including industrial production [3], agricultural cultivation [4], living environments [5], and human health [6]. During industrial processes, large quantities of flammable and explosive volatile compounds are frequently generated, presenting significant hazards to operational safety [7]. Concurrently, plants often release VOCs that function as pheromones during their growth cycles. These pheromones frequently serve as indicators of a plant’s physiological status and developmental progress [4]. The greenhouse effect and the depletion of the ozone layer are also closely related to the massive growth of VOCs [8]. Excessive inhalation of these volatile compounds constitutes a recognized health risk to humans [7]. To mitigate the adverse effects of volatile substances on normal production activities and daily life, diverse methodologies have been developed for VOCs concentration detection, including metal oxide sensors [9,10], conducting polymer sensors [11], gas chromatography [12], laser spectroscopy [13,14] and microwave-based sensing [15,16,17]. Among these, metal oxide gas sensors demonstrate favorable detection performance. However, their operational requirement for elevated temperatures imposes significant practical limitations [18]. Although conducting polymer gas sensors offer advantages such as compact size and ease of integration, they still face persistent challenges, including a complex fabrication process and susceptibility to environmental humidity, which await further research [19]. Gas chromatography and laser spectroscopy, despite its analytical precision, commonly relies on expensive instrumentation and complex procedures, which hinder its widespread practical deployment. In contrast, microwave sensing is a subject of active research for volatile gas detection, as it offers promising advantages such as low cost, easy integration, portability, and real-time detection [16,20].

Sensitivity, as a critical performance indicator for gas sensors, has consistently been a primary research focus. Currently, two principal approaches exist for enhancing the sensitivity of microwave gas sensors: utilizing superior gas-sensitive materials and designing novel microwave resonator structures. Promising gas-sensitive materials, such as polydimethylsiloxane (PDMS) [16], polymer-based (V_5_O_3_) beads [21] and PEDOT:PSS/MWCNT composites [22], have been successfully employed within microwave resonator configurations. However, some gas-sensitive materials often require complex manufacturing processes. Additionally, the performance of certain sensing materials may be susceptible to environmental interference [23], which could affect experimental results. Due to its low cost, ease of synthesis, and non-destructive reaction characteristics, PDMS is widely used in the field of volatile gas measurement. Microwave resonator structures are broadly categorized into planar resonators and cavity resonators. For planar resonators like split-ring resonators (SRR) [24,25,26,27], the sensing region is confined to the surface, failing to effectively utilize the strong electric field region constrained within the substrate. Consequently, their sensitivity is typically relatively low. Conversely, in cavity resonators, the analyte is exposed to the intense electric field within the cavity’s sensing volume, generally resulting in higher sensitivity. Moreover, various techniques aimed at miniaturization, gas flow optimization, and sensitivity enhancement have recently been reported. These include cavity folding [28], metal grid holes (METGH) [29], and the incorporation of air-filled layers [30]. Classical cavity resonator designs, such as RCR [28,31] and the substrate-integrated waveguide re-entrant cavity resonator (SIW RECR) [20,32,33], are widely utilized across diverse applications, including electromagnetic property characterization and humidity sensing, among other applications.

However, microwave sensors also exhibit inherent limitations. Due to the difficulty in directly observing radio frequency (RF) signals, microwave measurements typically rely on a VNA(Vector Network Analyzer) for signal acquisition. The VNA is a bulky and costly test instrument, hindering the development of portable or integrated microwave sensing applications. Multiport network can serve as a viable alternative to the VNA for RF signal measurement, enabling convenient and fast measurement because the measured vector values (such as phase and amplitude) can be converted into scalar power readings through a multiport network. Since the 1970s [34], multiport networks have been extensively studied and have demonstrated advantages including structural simplicity, high power efficiency, and low cost [32,35,36,37]. Furthermore, passive multiport networks configured using planar structures exhibit excellent robustness [38]. For sensing applications, a microwave sensor paired with an external signal source can be integrated as part of a self-contained microwave test system, connected to the multiport network. Such configurations are used to detect material dielectric properties [39], moisture content [37], angular displacement [40], and other parameters. During gas measurement processes, environmental conditions such as temperature and humidity frequently compromise measurement accuracy. In order to reduce the influence of external environmental factors on measurement results, adding reference sensors to the testing system is widely used. For microwave testing systems, on the one hand, couplers and power dividers can be used to load reference sensors into the network [41]. On the other hand, the same effect can be achieved by designing a differential cavity structure [20]. Both of these are applied to reduce the impact of environmental factors on measurements.

This paper presents the design of a testing system based on the RCR and a multiport demodulation network for the detection of volatile substances. Load the reference sensor and measurement sensor into the system through a ring coupler to form a sensing network. Detailed introduction of the design process and circuit principles of sensing elements. The relationship between the output phase and power detector voltage was determined using multiport demodulation network demodulation theory. Furthermore, the relationship between the output phase of the sensing network and the concentration of VOCs was determined through vector analysis. Finally, a real-time phase monitoring system based on LabVIEW was designed for real-time monitoring of volatile substances in gases. Theoretical analysis and experimental results verified the relationship between phase and VOCs concentration. Furthermore, verified that the system has the ability to suppress the influence of environmental factors.

**Figure 1:**
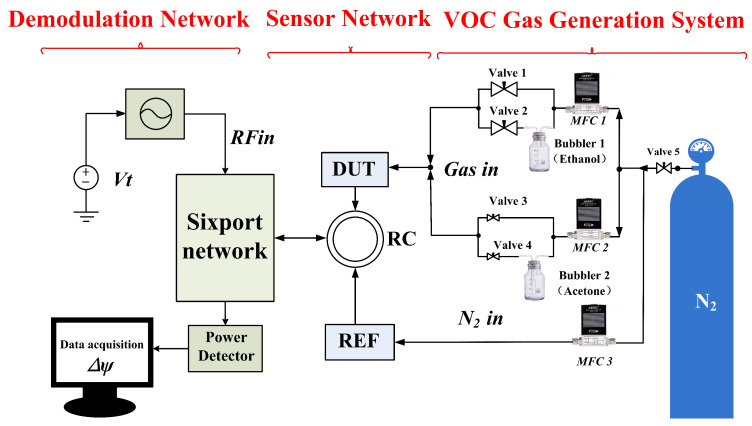
Diagram of sensor system for VOCs concentration detection based on RCR and multiport network.

Figure 1 presents the schematic diagram of the multiport network sensor structure for VOCs concentration measurement. The rectangular resonator, serving as the critical VOCs sensing element in this work, is embedded within the multiport network. To minimize the influence of external environmental factors on the test system, the reference sensor and measurement sensor are loaded into the system through a ring coupler to form a sensing network. To maximize the measurement sensor’s sensitivity, PDMS is loaded into its sensing region. Variations in VOC concentration induce shifts in the sensing element’s transmission phase due to changes in the equivalent distributed dielectric constant. The multiport network consists of a power divider, three hybrid networks, and a ring coupler. All components are equal power distribution components. Sensing information undergoes linear modulation by the multiport network before being fed into power detectors, which convert RF signals into DC voltage-represented power values. These power measurements are acquired in real-time by detectors and recorded via LabVIEW.Ultimately, the sensing phase angle is derived by analyzing multiple sets of LabVIEW-recorded power readings. This phase angle serves as the metric for characterizing VOCs concentration.

## 2. Theoretical Analysis of VOC Sensors Based on Multiport Network

### 2.1. Structural Design of RCR

**Figure 2:**
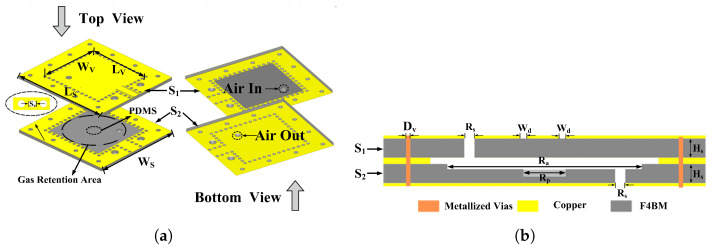
Geometric model of the RCR, where (**a**) shows the exploded view of the RCR and (**b**) shows the cross-sectional view of the RCR.

The VOCs sensor based on RCR is the core sensing element, and its structure is shown in Figure 2 and the device is composed of two layers of vertically stacked dielectric substrates, which are made of F4BM material with a relative dielectric constant of 2.65 and a loss tangent of 0.0013. Each F4BM substrate is covered with 0.035 mm copper foil on the front and back. In order to reduce the pollution of volatile substances to the environment during the test process, the sensor adopts the pump type measurement method. Substrate S1 and substrate S2, respectively, contain through holes with a radius of 0.8 mm for the entry and exit of test gas. In order to fully adsorb the test gas with PDMS, the air inlet of substrate S1 and the air outlet of substrate S2 are diagonally distributed. Furthermore, a gas retention area is left on the substrate S2. The top metal surface of substrate S1 is etched with Coplanar Waveguide (CPW) for signal transmission, as shown in Figure 2 a. Both substrates S1 and S2 contain metallized through holes to form a closed metal shell so that the electromagnetic field is limited inside the metal shell to avoid external radiation of the electromagnetic field. The cross-sectional view of RCR is shown in Figure 2b. Finally, the substrate S1 and S2 are tightened together through via arrays by use of screws to implement the electrical connections between them. The corresponding geometrical parameters of RCR are listed in Table 1.

### 2.2. Analysis of Reflection Response

The RCR can resonate at TE_*m*0*n*_ mode, and the corresponding resonant frequency can be determined as follows [28]:

(1) $$\;f_{{\mathrm{TE}}_{m0n}}=\frac{1}{2\sqrt{\mu_{\mathrm{eff}\;}\varepsilon_{\mathrm{eff}\;}}}\sqrt{{\left(\frac{m}{W_{v\;}}\right)}^{2}+{\left(\frac{n}{L_{v\;}}\right)}^{2}}$$

where *c*, $\mu_{\mathrm{eff}}$, $\varepsilon_{\mathrm{eff}}$, *m*, *n*, $W_{v}$, and $L_{v}$ are the light speed in vacuum, the effective permeability and permittivity of the substrate, the mode indices along the *x*- and *y*-axes, and the effective width and length of the RCR, respectively. Via array is used to implement the sidewalls of the RCR. Referring to (1), the RCR resonator with geometrical parameters listed in Table 1 theoretically demonstrates a resonant frequency of 8.31 GHz at TE_101_ mode. It is worth noting that when CPW is etched on the S1 surface, the resonant frequency point of RCR will change slightly. The working mechanism relies on the interaction within the sensor’s internal structure. A strong localized electric field is established within the sensor. When the gas passes through the strong E-field region in TE_101_ mode, PDMS undergoes physical adsorption with volatile substances. It causes the dielectric constant $\varepsilon_{\mathrm{eff}}$ inside the RCR to change, which will correspondingly change the resonant frequency point of TE_101_ mode obtained according to (1). This theory underpins our measurement of volatile gases employing the RCR technique. From the perspective of the circuit, it will cause a small change in the equivalent distributed capacitance C_*e*_ of LC resonant circuit in TE_101_ mode, as shown in the following equation:

(2) $$f=\frac{1}{2\pi \sqrt{L_{e}C_{e}}}$$

Since PDMS undergoes adsorption reactions with all volatile organic compounds (VOCs) to varying degrees, this sensor is specifically designed to study the impact of individual VOCs concentrations. This research focuses on acetone and ethanol as the target analytes.

### 2.3. Theory and Design of Six-Port Demodulation Networks

**Figure 3:**
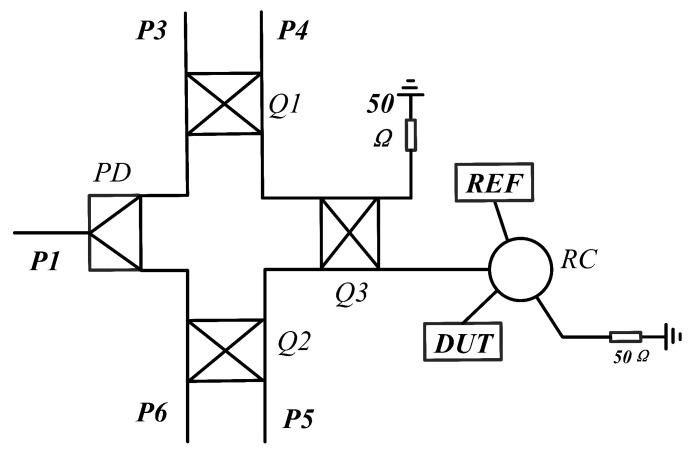
The structure of multiport demodulation circuit is composed of PD as Wilkins power divider, Q1, Q2, and Q3 as quadrature couplers, RC as ring coupler, and all devices are equal power distribution devices.

As shown in Figure 3, the demodulation circuit developed in this study comprises a Wilkinson power divider, three quadrature hybrid couplers, a ring coupler, and two RCR units. Both the reference (REF) and Device Under Test (DUT) sensors are integrated with the ring coupler. Note that the voltage signal extracted from the power detector after signal modulation enables phase information calculation through simple mathematical operations rather than direct frequency offset measurement.The multiport demodulation network is divided into a six-port demodulation network and a sensing network for analysis.

**Figure 4:**
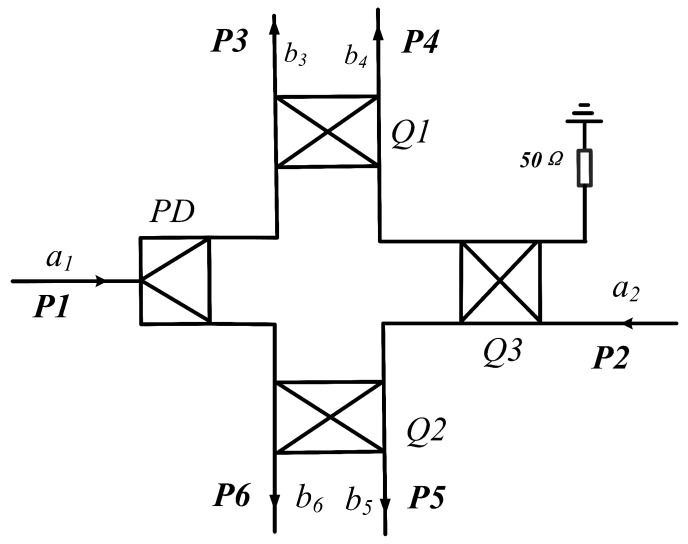
Schematic diagram of six-port demodulation network structure.

The designed six-port demodulation network structure is shown in Figure 4. The demodulation network consists of a power detector (PD) and three quadrature hybrid couplers (Q1, Q2, Q3). When an initial signal a1 is fed into the system, it flows through the six-port network toward port P2. If port P2 is terminated with a reflective sensor element, the resulting signal a2 carrying sensor data reflects into the six-port network. Through phase allocating by PD, Q1, Q2, and Q3, signals a1 and a2 subsequently undergo quadrature demodulation at ports P3, P4, P5, and P6.

The S-parameter matrix of the designed six-port network can be derived as follows [42]:

(3) $$\left[S\right]=\frac{1}{2}\left[\begin{array}{cccccc} 0 & 0 & 1 & -j & -j & 1\\ 0 & 0 & 1 & j & -1 & j\\ 1 & 1 & 0 & 0 & 0 & 0\\ -j & j & 0 & 0 & 0 & 0\\ -j & -1 & 0 & 0 & 0 & 0\\ 1 & j & 0 & 0 & 0 & 0 \end{array}\right]$$

Based on the S-parameter matrix of the six-port network (3), the relationship between $b_{3}$, $b_{4}$, $b_{5}$, $b_{6}$ and $a_{1}$, $a_{2}$ is given by:

(4) $$b_{i}=a_{1}S_{1i}+a_{2}S_{2i},\;i=3,4,5,6$$

In an ideal situation, the output voltage of the detector is proportional to the square of the input RF signal ($K_{0}$ and *K* are constants):

(5) $$V_{i}=K_{0}+K{\left|b_{i}\right|}^{2},$$

Expand $b_{i}$ according to the vector principle:

(6) $${\left|b_{i}\right|}^{2}=b_{i}^{2}=b_{i}\cdot b_{i}^{*},$$

Substituting Formula (4) into Formula(6) yields:

(7) $$|b_{i}{|}^{2}=a_{1}^{2}S_{1i}^{2}+a_{2}^{2}S_{2i}^{2}+a_{1}S_{1i}a_{2}^{*}S_{2i}^{*}+a_{1}^{*}S_{1i}^{*}a_{2}S_{2i},$$

This representation decomposes the complex symbol $a_{k}$ into its envelope $|a_{k}|$ and phase rotation factor $j\varphi_{k}$, thereby encoding modulation information in the frequency domain:

(8) $$a_{k}=\left|a_{k}\right|\cdot e^{j\varphi_{k}}\;k=1,2$$

Analogously, express $S_{ki}$ as a complex quantity:

(9) $$S_{ki}=\left|S_{ki}\right|\cdot e^{j\theta_{ki}}$$

For notational simplicity, we introduce:

(10) $$\begin{array}{c} \varphi_{2}-\varphi_{1}=\varphi \\ \theta_{2i}-\theta_{1i}=\theta_{i} \end{array}$$

Substituting (8)–(10) into (7) gives:

(11) $$|b_{i}{|}^{2}=\frac{|a_{1}{|}^{2}+|a_{2}{|}^{2}+2\left|a_{1}a_{2}\right|\cos\left(\varphi +\theta_{i}\right)}{4}$$

According to the Formula (3), $\theta_{i}$ can be obtained, Substituting (11) into (5) yields:

(12) $$\begin{array}{cc} I & =V_{3}-V_{4}=\frac{K|a_{1}a_{2}|\cos\varphi }{4}\\ Q & =V_{5}-V_{6}=\frac{K|a_{1}a_{2}|\sin\varphi }{4} \end{array}$$

Hence, the phase difference φ between input signals $a_{1}$ and $a_{2}$ is given by:

(13) $$\varphi =arctan\left(\left[\frac{Q}{I}\right]\right)=arctan\left(\frac{V_{5}-V_{6}}{V_{3}-V_{4}}\right)$$

Notably, although Formula (3) indicates complete isolation between ports P1 and P2 during signal $a_{1}$ excitation—theoretically preventing signal transmission from P1 to P2—practical measurements reveal non-zero $S_{21}$ parameters. This discrepancy implies measurable signal leakage from P1 to P2 in physical implementations.

### 2.4. Analysis of Sensor Networks

**Figure 5:**
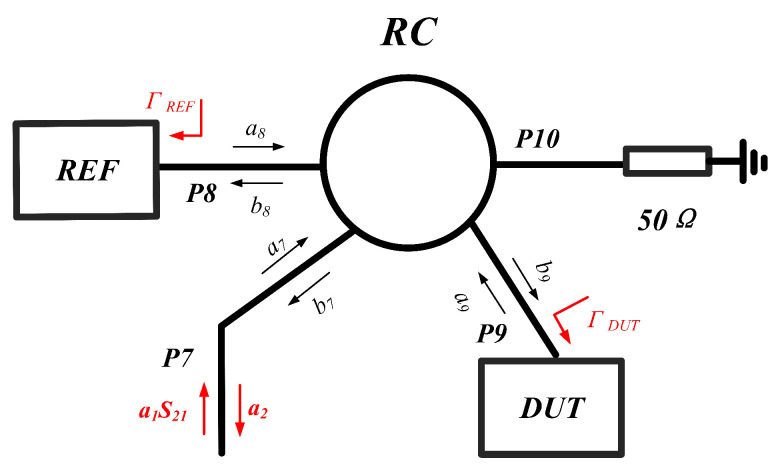
Schematic diagram of the rat-race coupler-based sensor configuration.

As derived above, the six-port network enables demodulation of the phase difference between $a_{1}$ and $a_{2}$. This facilitates measurement of VOCs by the RCR system. We now analyze the operating principle of the sensing network, which comprises a rat-race coupler (RC), REF, and DUT. The REF and DUT are positioned at the through and coupled ports of the rat-race coupler, respectively, with its structural configuration illustrated in Figure 5.

**Figure 6:**
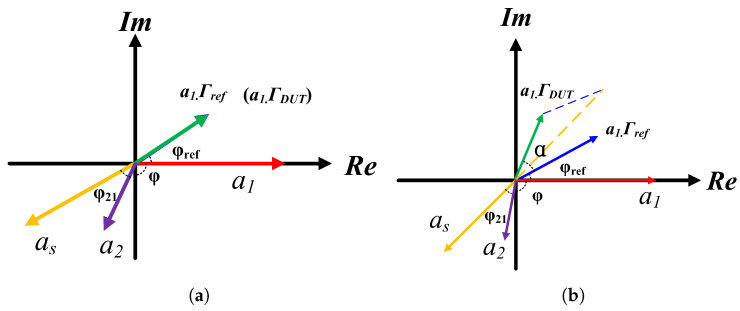
(**a**) Vector superposition process in the sensing network when both the REF and DUT are supplied with air (with no PDMS loaded in either REF or DUT at this stage) (**b**), vector superposition process in the sensing network when the DUT is supplied with VOC gases. Where $a_{s}$ is the vector superposition result of $-\frac{1}{2}\cdot a_{1}\cdot \left(\Gamma_{\mathrm{ref}}+\Gamma_{\mathrm{DUT}}\right)$.

When signal $a_{7}$ is input at port P7, RC equally splits the signal to ports P8 and P9. These ports are terminated with the REF and DUT sensors, respectively. Upon exposure to VOCs, the DUT induces a phase shift in the reflected signal propagating back into the RC, whereas the REF maintains a constant reference signal. Consequently, the resultant signal $a_{2}$ entering the six-port demodulation network satisfies the relationship:

(14) $$b_{7}=-\frac{j}{\sqrt{2}}\left(a_{8}+a_{9}\right)=-\frac{1}{2}\cdot a_{1}\cdot S_{21}\cdot \left(\Gamma_{ref}+\Gamma_{DUT}\right)$$

where $a_{1}$ is the initial input signal, $S_{21}$ is the transmission coefficient of the six-port demodulation network. $\Gamma_{\mathrm{ref}}$ and $\Gamma_{\mathrm{DUT}}$ are the reflection coefficients of REF and DUT, respectively, and $\varphi_{\mathrm{ref}}$ and $\varphi_{\mathrm{dut}}$ are the phases of the reflection coefficients. At this stage, the network enables measurement of the phase difference φ between the composite signal $\left(\Gamma_{\mathrm{ref}}+\Gamma_{\mathrm{DUT}}\right)$ and the original input signal $a_{1}$. However, the parameter of interest is the phase difference α between $\Gamma_{\mathrm{ref}}$ and $\Gamma_{\mathrm{DUT}}$. Consequently, we must establish the functional relationship α=f(φ). This derivation is facilitated through vector analysis as illustrated in Figure 6.

As illustrated in Figure 6a, when air is supplied to both the REF and DUT, their identical mechanical configurations produce identical reflections. The phase angle $\varphi_{1}$ is recorded at this stage using a power detector. By using VNA to detect multiport networks, $\varphi_{21}$ can be measured, so the angle $\varphi_{\mathrm{ref}}$ can be determined.

As depicted in Figure 6b, when VOCs is introduced into the DUT, the resulting alteration in its dielectric properties induces a phase shift. This creates a phase difference α between the signals $\Gamma_{\mathrm{ref}}$ and $\Gamma_{\mathrm{DUT}}$. Here, φ increases progressively with increasing α. Through geometric principles, it follows that:

(15) $$\tan\left(2\pi -\varphi -\varphi_{21}\right)=\frac{\sin\varphi_{\mathrm{ref}}+k\sin\left(\varphi_{\mathrm{ref}}+\alpha \right)}{\cos\varphi_{\mathrm{ref}}+k\cos\left(\varphi_{\mathrm{ref}}+\alpha \right)}\;\mathrm{where}\;k=\frac{A_{\mathrm{ref}}}{A_{\mathrm{DUT}}}$$

**Figure 7:**
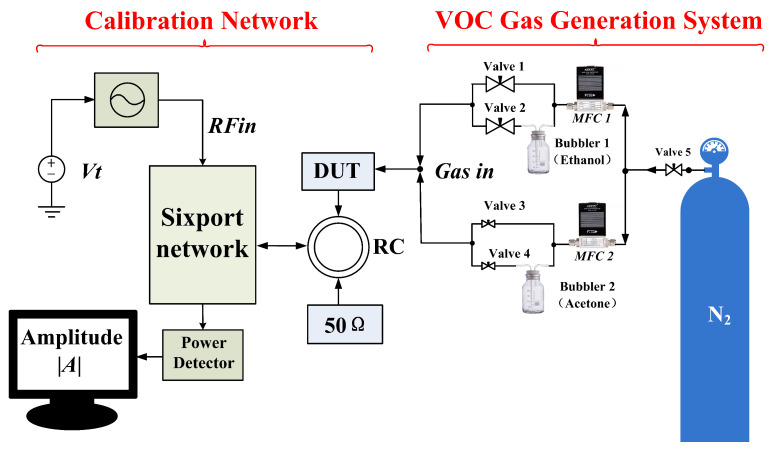
The flow chart of the calibration system.

From Equation (15), it can be seen that the relationship between φ and α is influenced by the value of *k*. In order to eliminate the effect of the *k* value on the test results, a calibration platform as shown in Figure 7 is established for system calibration. The calibration formulas are as follows:

(16) $$V_{DUT}=k_{1}b_{10}^{2}=\frac{k_{1}}{4}|S_{21}{|}^{2}\cdot {\left|A_{DUT}\right|}^{2}.a_{1}^{2}$$

Through sequential measurements introducing both ambient air and VOC vapors at graded concentrations, the corresponding *k*-values for each dataset during continuous testing can be determined (when both REF and DUT are supplied with air, $A_{\mathrm{dut}}=A_{\mathrm{ref}}$).

## 3. Description of the Test Platform

The VOC gas generation system is an important component of the testing process.Within the VOC generation system, the Volatile Organic Compounds (VOCs)—namely ethanol and acetone in this work—are obtained by evaporating their respective liquid solutions. Ethanol and acetone solutions are separately housed in washing bottles 1 and 2. Nitrogen gas ($N_{2}$) serves as the carrier gas to dilute the VOC concentration. The volumetric flow rate in each of the two channels is initially regulated by two mass flow controllers (MFC). Subsequently, four valves are employed to open or close the four individual channels, enabling the generation of various concentrations of ethanol and acetone. For instance:When valves 1 and 4 are closed and consequently valves 2 and 3 are open, the MFC flow rates can be adjusted to yield different concentration levels of ethanol. Conversely, with valves 2 and 3 closed and valves 1 and 4 open, varying concentrations of acetone can be produced.The generated VOC mixture is then pumped into the sensor module via its inlet port located at the top for detection.This phase is designated as the adsorption process.Following the completion of the adsorption process, valves 2 and 4 are closed, and valves 1 and 3 are opened. Pure $N_{2}$ is pumped into the sensor, facilitating its reversion to the initial state in preparation for subsequent detection cycles. The specific gas flow rates are set as shown in Table 2.

Due to its ability to adsorb volatile organic compounds (VOCs) and subsequently desorb them when exposed to air, PDMS is recognized as an effective sensitive material for VOC detection. Upon exposure to VOCs, PDMS undergoes a swelling effect, leading to a measurable change in its relative permittivity. The extent of PDMS swelling is governed by the concentration of the VOCs present. Consequently, depositing PDMS within the sensing region of an RCR structure significantly enhances the sensor’s sensitivity to VOCs [16,20]. HFSS simulation analysis indicates that the thickness of the PDMS layer influences the reflection coefficient of the RCR sensor. To ensure optimal performance of the RCR structure, a PDMS thickness of 0.2 mm was selected. A volume of 1.5 mL of liquid PDMS was used to form the PDMS film with the specified thickness. To obtain solid PDMS at room temperature, the liquid PDMS prepolymer was uniformly mixed with its curing agent at a mass ratio of 1:10. The resulting mixture was then allowed to sit for 15 min to facilitate the removal of small air bubbles entrapped during stirring. Subsequently, a controlled volume of the degassed mixture was pipetted onto the sensing area of substrate S2 and spin-coated to ensure a uniform film thickness. Finally, the substrate S2 bearing the deposited PDMS film was placed on a hot plate at 100 °C for 20 min. This thermal curing step promoted the formation of a cross-linked polymer network, resulting in the complete solidification of the PDMS layer.

**Figure 8:**
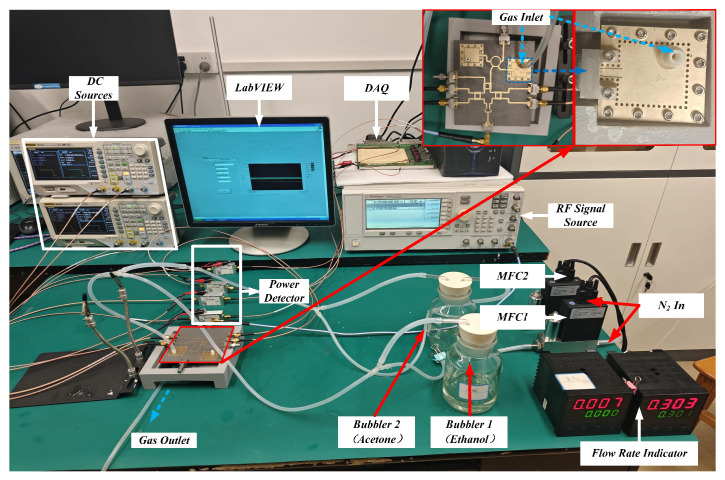
Physical picture of volatile testing system.

Figure 8 depicts the setup of the testing system. The target gas is generated by a gas generator and is directed into the sensor via a flexible tube. As the gas concentration changes, the sensor’s reflective signal varies.The reference sensor is a key component of the experimental setup. The reference and measurement sensors are both RCR and share an identical mechanical structure; however, the reference sensor is not coated with PDMS. During measurement, both sensors are placed in the same environment. The reference sensor is exposed to ambient air, while the target gas is delivered to the measurement sensor via a gas generation system. This signal is then transmitted through the sensing network to a six-port demodulation circuit. Ports P3, P4, P5, and P6 of the multiport demodulation network are connected to power detectors for signal measurement. The data acquisition card (DAQ) coupled with LabVIEW (Version 2018) enables real-time monitoring of data variations throughout the adsorption process. The power detectors are powered using a 5 V supply voltage. After undergoing simple filtering and calculation, the acquired data is processed using MATLAB (Version R2023 a). This processing yields the final relationship between phase angle α and VOC concentration. During measurements of different gases, the input signal power from the source was maintained constant at −15 dBm to ensure the transmission signal remained within the optimal operating range of the power detector. Test frequencies were selected as follows: 8.15 GHz for acetone and 8.175 GHz for ethanol. This is due to the subtle differences between integrated circuits and components.

## 4. Measurement Results and Analysis

### 4.1. Component Functionality Testing

**Figure 9:**
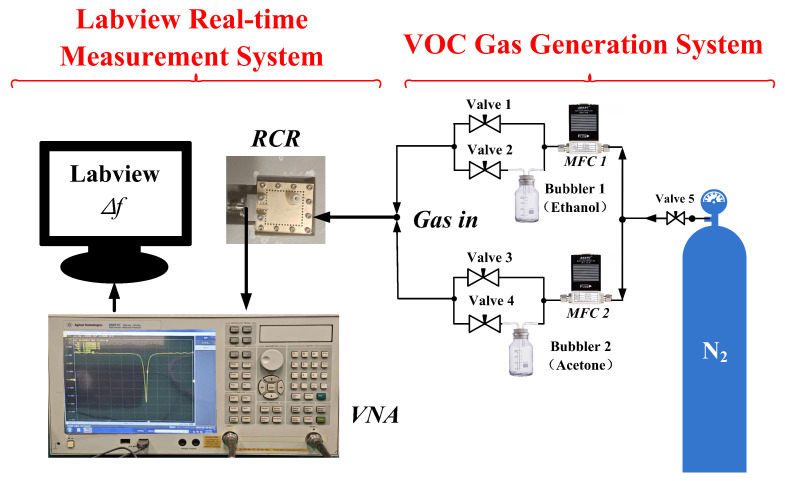
Flow chart of RCR sensor function verification system.

A set of test platforms is built to verify the above design process. The test system flow chart is shown in Figure 9, in which RCR is directly connected to a VNA through a coaxial cable. The incoming and outgoing pagoda joints are 3D printed. The pagoda joints is 3D printed from PLA (polylactic acid) and tightly connected to the substrate with sealant. Due to manufacturing tolerances and welded joint losses, the actual resonant frequency slightly deviates from the theoretical value.

**Figure 10:**
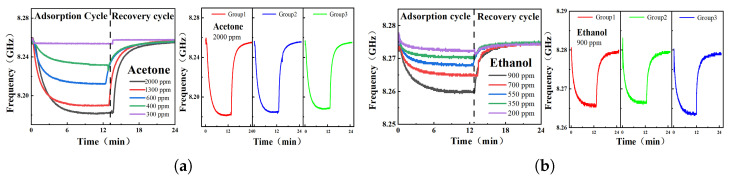
Real-time oscillation frequency variation test graph of RCR exposed to different concentrations of VOC gas. In (**a**), the measured gas is acetone, and in (**b**), the measured gas is ethanol.

When the sensing area changes from air (unloaded) to loaded PDMS, the resonant frequency point will decrease due to the increase in dielectric constant. The theory is based on (1). Subsequently, Figure 10 illustrates the frequency shifts of the RCR under varying concentrations of acetone and ethanol gases. Given ethanol’s significantly lower volatility than acetone, distinct total gas flow rates were applied to achieve target ethanol concentrations: 600 mL/min for ethanol testing versus 300 mL/min for acetone testing. Consequently, the initial resonance frequencies differed slightly between these two test conditions. As shown in Figure 10a,b, LabVIEW real-time recordings capture the RCR’s frequency shift during acetone (ethanol) exposure. When the RCR is in the environment of acetone (ethanol) with different concentrations, the resonance frequency shifts to different degrees. The deviation is proportional to the concentration of acetone (ethanol). This is because the higher the concentration of VOCs, the greater the adsorption degree of PDMS. A maximum frequency shift of 78 MHz was observed in RCR under acetone saturation.

Through the above experiments, the designed RCR demonstrates its capability to quantify varying concentrations of VOCs. However, practical deployment remains challenging due to its reliance on an expensive VNA. Therefore, solutions for signal detection have become crucial. The proposed demodulation network provides a feasible alternative solution for measurement.

**Figure 11:**
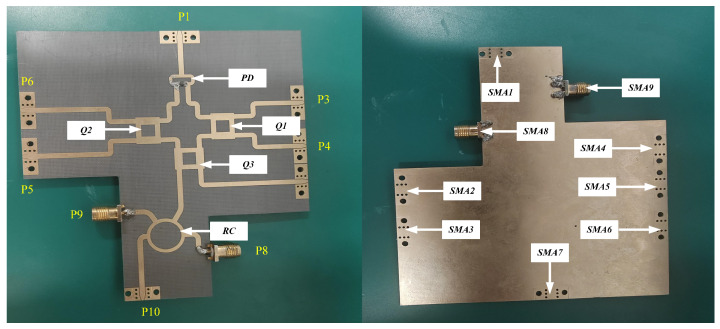
Material object of multiport network.

**Figure 12:**
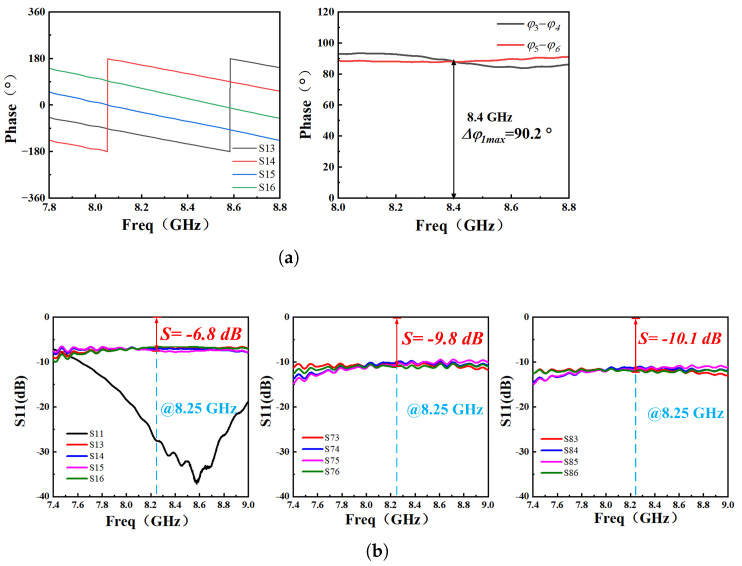
The performance parameter test results of the multiport network, where (**a**) is the phase parameter test result of the multiport network, and (**b**) is the reflection coefficient test result of the multiport network.

Given that the designed multiport demodulation network is an integrated structure, it was imperative to evaluate the functionality of both the six-port demodulation network and the rat-race coupler prior to conducting tests. Figure 11 shows the physical implementation of the six-port demodulation network and the rat-race coupler. This network was characterized using a VNA. The corresponding measurement results are illustrated in Figure 12. Within the operational frequency band of 8.1 GHz to 8.5 GHz, the phase differences between respective ports align with theoretical expectations.

However, due to substrate material properties and design tolerances, the reflection coefficients of Ports P3/P4 and P5/P6 exhibit minor deviations from theoretical values. This discrepancy does not compromise the overall demodulation functionality of the network. To mitigate potential impacts from excessive assembly and soldering on the test system integrity, all components were interconnected using microstrip lines in subsequent testing phases. This configuration forms an integrated RF transmission path, with the exception of the REF and DUT substrates (designated S2), which are mechanically fastened to the main substrate using screws.

### 4.2. Phase Response of VOCs

The test system was configured according to Figure 8 for acetone and ethanol concentration testing. During testing, Valves 1 and 3 were initially opened to purge the gas line with nitrogen. Following system calibration, LabVIEW was activated for real-time data acquisition while executing gas switching. Maintain continuous gas flow through the test section with a 15-min sorption phase (gas exposure) and a 10-min desorption phase (recovery in nitrogen ambient).To ensure experimental reproducibility and accuracy, The gas concentrations generated by the gas supply apparatus were systematically calibrated, and three sets of repetitive tests were conducted.

**Figure 13:**
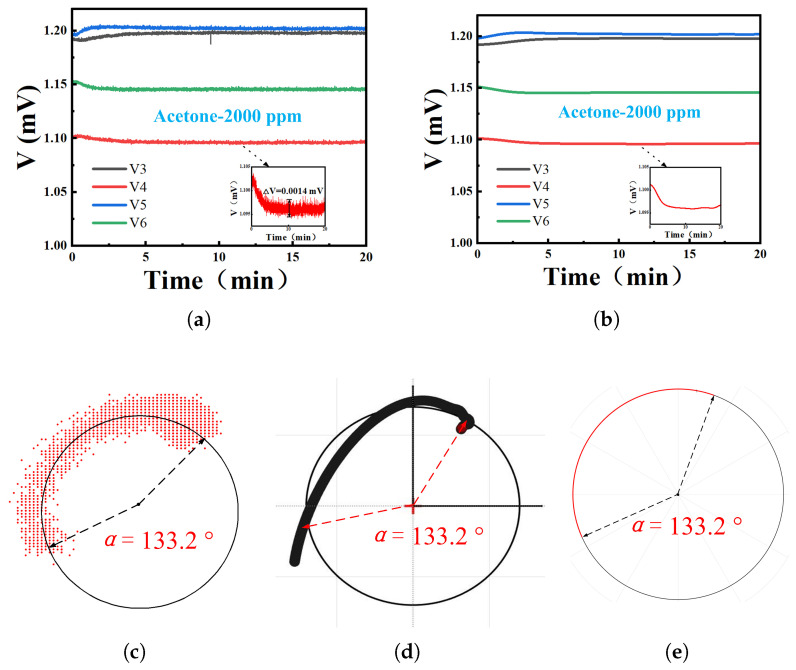
Comprehensive Signal Demodulation Workflow with LabVIEW. Among them, (**a**) shows the real-time data collected by LabVIEW, (**b**) shows the filtered data collected in real-time, (**c**) shows the demodulation result of unfiltered data, (**d**) shows the demodulation result of filtered data, and (**e**) shows the filtered data after being filtered again by software Matlab2023a.

To elucidate the phase demodulation mechanism of this system, we analyze the 2000 ppm acetone measurement as a representative case. Figure 13a displays the voltage data recorded by LabVIEW during testing, revealing distinct voltage shifts upon VOC introduction. This phenomenon originates from the increased dielectric constant of the PDMS within the RCR due to gas adsorption. Subsequent voltage stabilization corresponds to PDMS adsorption saturation, where dielectric constant variation ceases. As evidenced by Figure 10 and Figure 13a, the detection limits for acetone and ethanol are 300 ppm and 200 ppm, respectively. Below these thresholds, gas-induced voltage deviations become indistinguishable from system noise. Inherent noise from the power detector and demodulation network necessitated signal filtering (Figure 13b). To mitigate the influence of inherent noise from the power detector and sensor system on the acquired data, an FFT filtering technique implemented in OriginPro 2024 (OriginLab Corporation). was employed to effectively reduce its impact on the measurement results. The processed data were then input into a custom MATLAB program implementing Equation (13), yielding the phase-demodulated results in Figure 13d. For verification, unfiltered data produced Figure 13c, showing points distributed along a circular arc with angular consistency to filtered results. To enhance measurement visualization, we optimized the code architecture to constrain the curve onto a unified circle (Figure 13e), enabling direct phase angle quantification.

**Figure 14:**
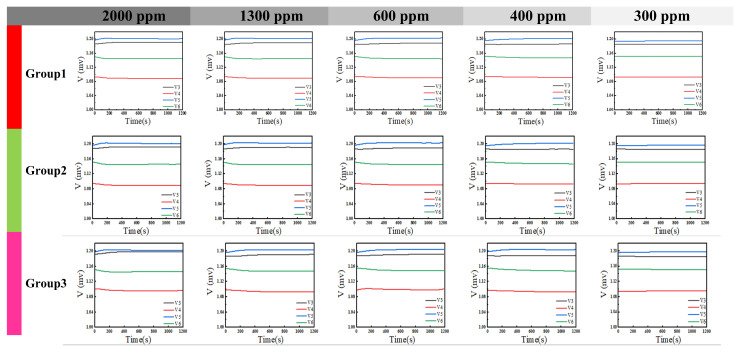
Filtered voltage response to acetone vapor via LabVIEW.

**Figure 15:**
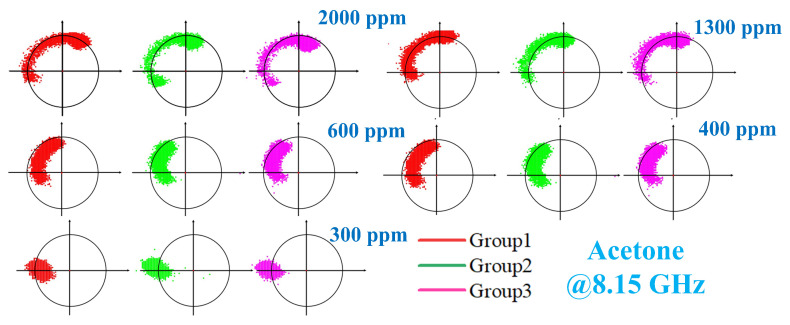
Test results after signal demodulation of unfiltered data for detecting different concentrations of acetone.

**Figure 16:**
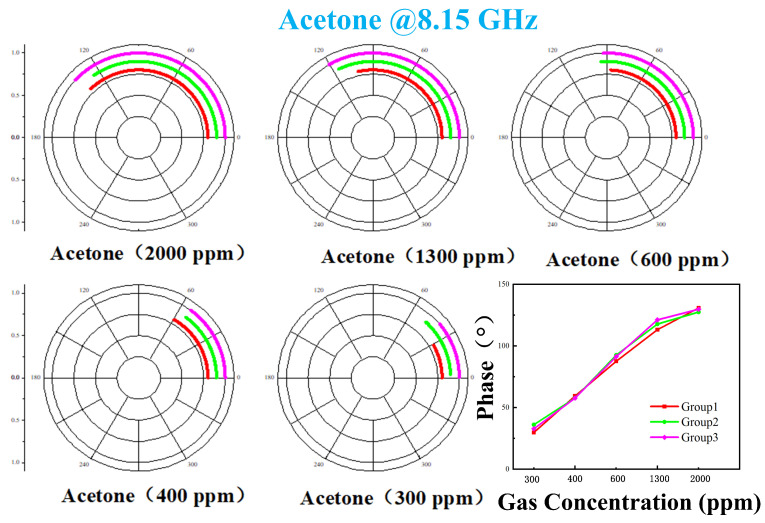
When detecting different concentrations of acetone, the filtered data is demodulated and the test results are obtained.

Acetone gas samples at various tested concentrations were subjected to the identical procedure described above. The voltage data recorded by LabVIEW, after filtering, are presented in Figure 14. It is evident that as the gas concentration increased, the corresponding change in output voltage exhibited a stronger response. Unfiltered data were processed using a MATLAB algorithm, with the results shown in Figure 15. All data points are distributed in proximity to an arc corresponding to phase variations. Furthermore, lower concentrations resulted in smaller phase change magnitudes and more densely clustered data points. Subsequently, the filtered data were demodulated using MATLAB code, yielding the results shown in Figure 16. Figure 16 demonstrates that higher gas concentrations produced larger demodulated phase values from the system, indicating a positive correlation between concentration and phase shift.

**Figure 17:**
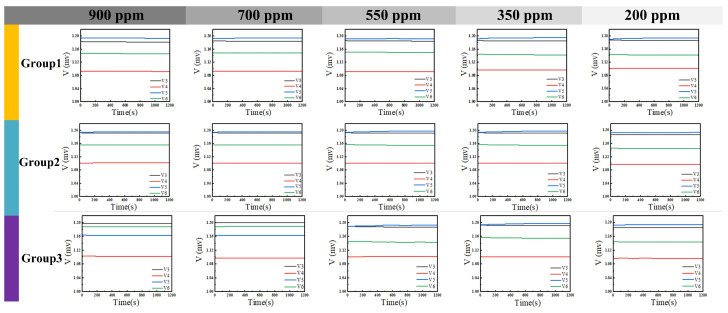
Filtered voltage response to ethanol vapor via LabVIEW.

**Figure 18:**
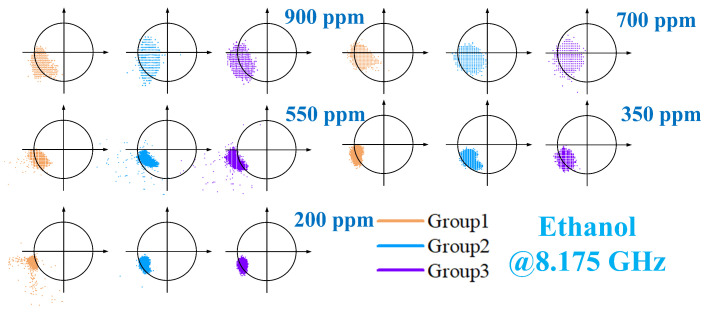
Test results after signal demodulation of unfiltered data for detecting different concentrations of ethanol.

**Figure 19:**
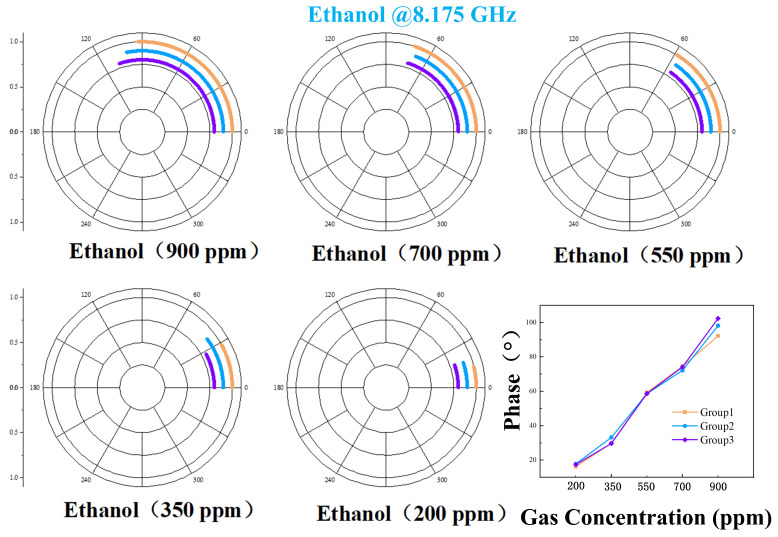
When detecting different concentrations of ethanol, the filtered data is demodulated and the test results are obtained.

Minor structural perturbations within the Resonant Cavity (RCR), attributable to fluctuations in carrier gas flow rate, led to a discernible shift in its fundamental resonance frequency. To compensate for this effect and maintain optimal sensitivity, the operating frequency for ethanol vapor detection was precisely tuned to 8.175 GHz. Ethanol test data, recorded via LabVIEW, were processed using an identical methodology to that established for acetone analysis. Graphical results spanning from Figure 17, Figure 18 and Figure 19 confirm that ethanol concentration trends mirror those previously observed for acetone: a pronounced increase in measured phase angle correlates directly with rising vapor concentrations. Rigorous validation of result reliability was implemented. LabVIEW facilitated continuous, real-time voltage acquisition at the demodulation output ports, while triplicate independent measurements were performed for each condition. Comparative analysis of the IQ trajectories in Figure 16 and Figure 19 reveals minimal deviation between replicate datasets, unequivocally demonstrating the system’s excellent measurement repeatability for both analytes.

### 4.3. Temperature and Humidity Effects

**Figure 20:**
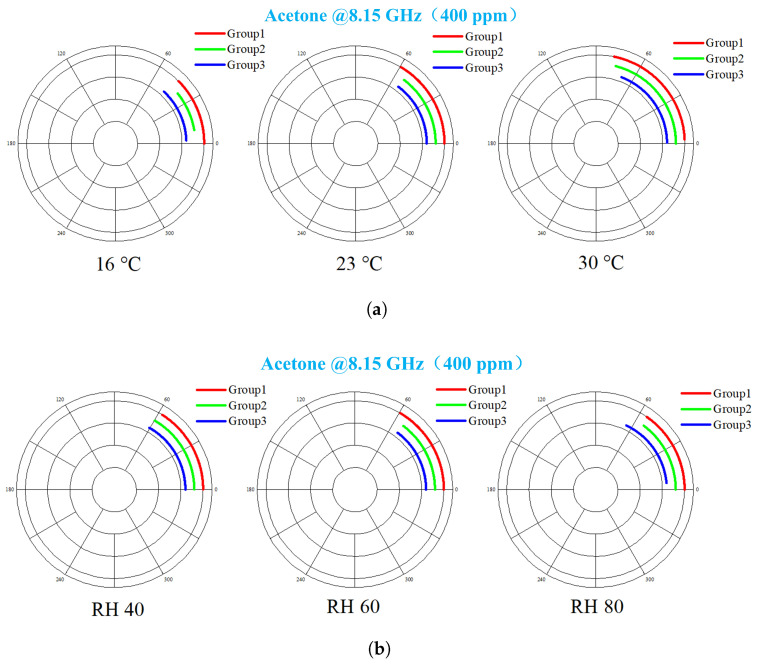
Measurement results of acetone under different temperature and humidity environments, where (**a**) is the temperature-dependent acetone test results and (**b**) is the humidity-dependent acetone test results.

Measurement deviations in gas sensing systems stemming from temperature and humidity fluctuations pose significant challenges. To counteract this environmental interference, the current system employs a reference-sensor compensation strategy, schematically illustrated in Figure 6. The key principle relies on the structurally identical reference and measurement sensors experiencing highly correlated drift under variations in ambient temperature or humidity. Thus, the impact of environmental changes is effectively isolated to the $\varphi_{\mathrm{ref}}$ parameter, substantially boosting measurement accuracy. Experimental evaluation utilized 400 ppm acetone vapor across controlled thermal–hygrometric gradients. As demonstrated in Figure 20a, concentration readings increase with temperature. This behavior arises from the temperature-dependent vapor pressure elevation in the isochorically contained acetone-water binary system, consistent with the Clapeyron relation. Higher temperatures increase the actual acetone partial pressure/concentration, leading to stronger phase shift responses. Humidity test results (Figure 20b) reveal the system exhibits remarkably low sensitivity to changes in humidity level. Despite the significant dielectric constant difference between nitrogen background gas and water vapor molecules, increasing the mixture’s permittivity (which theoretically could cause substantial resonance perturbations), the reference compensation mechanism successfully cancels this effect. System robustness against environmental perturbations was validated through triplicate trials, confirming high repeatability.

### 4.4. Work Comparison

As benchmarked in Table 3, this work is compared against existing technologies for VOC detection, while some referenced sensors demonstrate high sensitivity, their performance is critically dependent on specific operating temperatures, which often deviate from ambient conditions and thus limit their practical applicability. In contrast, the system proposed in this work offers distinct advantages. It achieves a competitive detection limit while ensuring operational robustness. The capability for rapid and user-friendly measurements is a key feature. Moreover, the design incorporates effective measures to mitigate the confounding effects of temperature and humidity fluctuations, thereby enhancing the reliability of the results in real-world testing scenarios.

## 5. Conclusions

In this work, a novel multiport demodulation system is developed for the precise measurement of volatile compound concentrations within gaseous samples, offering marked improvements in stability. Central to this innovation is the integration of dual RCR elements within the demodulation circuit core. A rat-race coupler is employed in an innovative configuration to simultaneously inject both reference and sensing signals into the multiport junction. This design strategy functionally emulates the core advantages of a differential architecture, thereby significantly enhancing the stability of the measurement platform. The critical phase information generated by this process is accurately converted into a measurable power output characterized by voltage, which serves as the fundamental input for estimating volatile analyte concentration. Comprehensive theoretical analysis substantiated the system’s feasibility. This included rigorous scrutiny of the demodulation circuit’s operational principle and the pivotal role played by the rat-race coupler. Based on multiport network theory, we derived and exhaustively characterized a well-defined quantitative relationship between the output phase shift and the resulting power output. Experimental verification was performed using a custom gas generation platform integrated with a LabVIEW-controlled real-time detection system. Findings confirm the system’s ability to reliably quantify VOCs concentrations via high-precision phase measurements, demonstrating well-detection sensitivity. Importantly, the architecture exhibits robust mitigation of measurement errors induced by ambient temperature and humidity variations—a key practical benefit for gas sensing applications. An additional significant environmental advantage stems from the use of polydimethylsiloxane (PDMS) as the sensing material. Interaction between PDMS and VOCs occurs solely via reversible physical adsorption mechanisms. This process ensures the sample gas’s chemical composition remains unaltered and permits straightforward, contamination-free sensor regeneration through simple air purging cycles. This intrinsic operational mechanism endows the system with high environmental compatibility and non-polluting characteristics.

## Figures and Tables

**Table 1:** Geometric Dimensions of RCR.

Para.	Description	Value (mm)
*Ls*	Substrate length	29
*Ws*	Substrate width	26
*Hs*	Substrate thickness	0.8
*Rs*	Inlet and outlet holes	0.8
*Sv*	Metallic via spacing	1.35
*Dv*	Metallic via size	0.45
*Ra*	Radius of retention area	8
*Rp*	PDMS loading area radius	2
*Wd*	CPW width	0.25
*Lv*	Length of cavity	17.4
*Wv*	Cavity width	16.3

**Table 2:** Test gas concentration parameters.

Acetone		Ethanol	
Total Flow: 300 mL/min		Total Flow: 600 mL/min	
Ratio	Conc. (ppm)	Ratio	Conc. (ppm)
5:0	2000	6:0	900
4:1	1300	5:1	700
3:2	600	4:2	550
2:3	400	3:3	350
1:4	300	2:4	200

**Table 3:** Comparison between this work and other VOC sensors.

Ref.	Working Environment	Sensitive Materials	Detection Limit (ppm)	Environmental Robustness	Portable Type
[10]	100 °C	Agup	15.4 (acetone)	No	No
[15]	RT	PGMA/POEGM	270 (acetone) 600 (ethanol)	No	No
[39]	RT	Comb polymer Pc-based thin film	0.5 (acetone)	No	Yes
[17]	RT	Customized P25DMA	625 (acetone)	No	No
T.W.	RT	PDMS	300 (acetone) 200 (ethanol)	Yes	Yes

## Data Availability

The data used to support the findings of this study are available from the corresponding author upon request.

## Abbreviations

The following abbreviations are used in this manuscript:

VOC	Volatile Organic Compounds
RCR	Rectangular Cavity Resonators
PDMS	Polydimethylsiloxane
RF	Radio Frequency
VNA	Vector Network Analyzer
CPW	Coplanar Waveguide
REF	Reference
DUT	Device Under Test
SIW	Substrate-Integrated Waveguide
RECR	Re-entrant Cavity Resonator
TE	Transverse Electric
PD	Power Divider
RC	Rat-race Coupler
MFC	Mass Flow Controller
DAQ	Data Acquisition
SRR	Split-Ring Resonators
METGH	Metal Grid Holes
